# Multidrug resistance and genomic characteristics of nontypeable *Haemophilus influenzae* isolates from the respiratory tract of pediatric patients

**DOI:** 10.1128/spectrum.00498-26

**Published:** 2026-08-14

**Authors:** Zhuyuan Wen, Yiming Kong, Mengyao Ling, Jingyuan Zhang, Bing Du, Zihui Yu, Chao Yan, Guanhua Xue, Lin Gan, Junxia Feng, Zheng Fan, Tongtong Fu, Ziying Xu, Yang Yang, Tingting Zhang, Yanling Feng, Hanqing Zhao, Jinghua Cui, Jing Yuan

**Affiliations:** 1 Department of Bacteriology, Capital Center for Children’s Health, Capital Medical University, Capital Institute of Pediatrics 36776, Beijing, China; Griffith University - Gold Coast Campus Institute for Glycomics, Gold Coast, Australia

**Keywords:** nontypeable *Haemophilus influenzae *(NTHi), multidrug resistance (MDR), genome analysis, children, sputum, bronchoalveolar lavage fluid (BALF)

## Abstract

Nontypeable *Haemophilus influenzae* (NTHi) is a common colonizer of the human upper respiratory tract and one of the major pathogens responsible for pediatric respiratory tract infections. Given the increasing severity of its multidrug resistance (MDR), this study comprehensively investigated the genomic characteristics of circulating NTHi isolated from sputum and bronchoalveolar lavage fluid (BALF). A total of 104 *H. influenzae* isolates (69 from sputum; 35 from BALF) were collected from pediatric patients between January 2024 and January 2025. All isolates underwent whole-genome sequencing and antimicrobial susceptibility testing, followed by core/pan-genome phylogenetic analysis, multilocus sequence typing (MLST), and resistome profiling. Among them, 103 were identified as NTHi. We identified 29 known sequence types (STs) and 10 novel STs, with ST-107 (14.4%), ST-57 (10.6%), and ST-11 (8.7%) being the major circulating lineages. However, core-genome phylogenetic analysis provided a more granular view of the genetic variation within these identical STs. All the isolates showed high resistance to ampicillin (98.1%) and cefuroxime (84.6%). Genomically, the multidrug efflux pump gene *hmrM* was ubiquitous (100%). Ampicillin resistance was predominantly driven by *blaTEM-1* carriage (77.9%), with minor contributions from chromosomal *ftsI* mutations. Fifteen plasmid replicons were predicted from 25 isolates, which highly coincided with the carriage of *blaTEM-1* and other acquired resistance genes. This study demonstrates that MDR in pediatric NTHi is primarily driven by acquired resistance genes and chromosomal mutations, with specific resistant clones persisting and enriching under clinical antibiotic pressures. These findings underscore the importance of continuous high-resolution genomic surveillance in guiding rational antibiotic stewardship.

**IMPORTANCE:**

This study highlights the critical importance of high-resolution genomic surveillance in managing pediatric nontypeable *Haemophilus influenzae* (NTHi) infections. By utilizing whole-genome sequencing, we uncovered the pathogen’s highly dynamic population structure and complex multidrug resistance (MDR) mechanisms. Crucially, our findings reveal a strong, non-random coupling between core genomic architectures, virulence factors, and MDR elements, driven by dual environmental and pharmacological pressures. This “virulence-MDR” co-evolutionary trend underscores the persistent clinical threat of locally adapted high-risk clones. These findings provide important insights for guiding rational clinical antibiotic stewardship, optimizing treatment strategies, and improving regional infection control.

## INTRODUCTION

*Haemophilus influenzae* (Hi) is a Gram-negative bacterium that colonizes the respiratory tract in up to 80% of healthy children (1, 2). Hi includes both encapsulated and unencapsulated strains, with encapsulated strains classified into six serotypes (Hia–Hif). Among them, *H. influenzae* type b (Hib) has historically been regarded as the most virulent (3, 4). The widespread application of Hib vaccination has led to a substantial reduction in infections caused by Hib (5, 6). According to Global Burden of Disease data (7), Hib pneumonia, respiratory syncytial virus pneumonia, pneumococcal pneumonia, and influenza were the leading causes of lower respiratory tract infection-related deaths in children under 5 years of age.

With the increasing prevalence of multidrug resistance (MDR) nontypeable *H. influenzae* (NTHi), elucidating the concordance between phenotypes and genotypes is of paramount importance. Previous studies indicate that MDR in NTHi is primarily driven by a combination of specific resistance genes, including *blaTEM-1*, *mef(A*), and *msr(D*), alongside chromosomal target alterations, such as *ftsI* mutations (8, 9). For epidemiological tracking, traditional approaches have extensively relied on multilocus sequence typing (MLST) (9–11). For instance, previous surveillance in China has revealed significant geographical heterogeneity, identifying regional prevalent lineages, such as ST-107, in Shanghai and Guiyang (9–11), ST-408/ST-914 in Beijing/Chongqing (10), and ST-12 in Guangzhou (12). However, given the extreme genetic heterogeneity and frequent homologous recombination inherent to NTHi, sequence typing based on merely seven housekeeping genes often lacks sufficient discriminatory resolution to accurately trace clonal dissemination and the dynamic evolution of resistance determinants (6, 13). Recently, core-genome typing derived from whole-genome sequencing (WGS) has demonstrated ultra-high resolution. Compared to traditional MLST, WGS-based analysis precisely clusters identical STs into monophyletic clades to reveal deeper intra-lineage diversity, enabling a more precise delineation of NTHi population dynamics and the horizontal transfer of resistance elements (6).

In clinical settings, Hi is primarily isolated from sputum or bronchoalveolar lavage fluid (BALF); however, previous studies have rarely systematically compared and differentiated these two sample types utilizing high-resolution genomics. In fact, these two sample types reflect fundamentally distinct host states and selective pressures: sputum predominantly represents newly diagnosed patients with relatively mild symptoms, whereas BALF is typically obtained from patients with refractory infections who have experienced repeated and high-intensity empirical antibiotic treatment failures (14, 15). Intensive clinical antibiotic exposure is widely recognized as a critical driver of bacterial evolution. However, despite the distinct selective pressures associated with different sample sources, comprehensive large-scale analyses utilizing high-resolution genomics are still lacking, limiting our ability to systematically evaluate how these divergent microecological environments shape the phenotypic and genomic characteristics of NTHi (14).

To address these clinical challenges, we comprehensively analyzed a cohort of MDR NTHi isolates recovered from pediatric respiratory tracts. Using WGS, we constructed a high-resolution core-genome phylogeny to trace the evolutionary trajectories of predominant clones. Furthermore, we systematically integrated predicted resistance genotypes with actual antimicrobial susceptibility testing (AST) phenotypes. By linking these genomic and phenotypic profiles to real-world clinical metadata (specimen sources and prior antibiotic exposure), this study aims to elucidate how clinical selective pressures shape the genomic diversity, virulence potential, and MDR patterns of NTHi. Ultimately, our findings provide a critical foundation for precision management of severe pediatric respiratory infections.

## RESULTS

### Clinical characteristics of the study cohort

A total of 104 Hi strains were isolated from sputum (*n* = 69) and BALF (*n* = 35) of hospitalized children between January 2024 and January 2025. All isolates were initially identified by matrix-assisted laser desorption/ionization time-of-flight mass spectrometry (MALDI-TOF MS). One patient contributed both sputum and BALF samples simultaneously. The patients ranged in age from 0 to 15 years: 36.9% (38/103) were younger than 1 year, 24.3% (25/103) were 1–5 years, 31.1% (32/103) were 5–10 years, and 7.8% (8/103) were 10–15 years. Of the 103 patients, 56.3% (58/103) were male and 43.7% (45/103) were female. Based on clinical diagnoses, the cases were classified as bacterial pneumonia (9.7%, 10/103), viral pneumonia (7.8%, 8/103), Mycoplasma pneumoniae (5.8%, 6/103), mixed pneumonia (65%, 67/103), and other non-respiratory diseases (11.7%, 12/103). The detailed information is listed in Table S1.

Furthermore, to account for potential host-related confounding factors, a retrospective comparison of prior pharmacological exposures was conducted between patients with sputum isolates and those with BALF isolates (Table 1). Although the overall rates of prior antibiotic exposure were comparable between the two cohorts, their specific exposure patterns exhibited highly significant differences (*P <* 0.001). Notably, 80.0% of the patients in the BALF group had a history of prolonged or repeated antibiotic exposure, compared with only 23.2% in the sputum group. Regarding specific antimicrobial classes, the prior administration of macrolides and tetracyclines was significantly more prevalent in the BALF cohort (*P =* 0.017 and *P <* 0.001, respectively). These clinical characteristics strongly suggest that the isolates from BALF and sputum were subjected to fundamentally different intensities of pharmacological selection pressure prior to sampling.

**TABLE 1:** Prior pharmacological exposures of patients before sample collection

Characteristics	Sputum (*n* = 69)	BALF (*n* = 35)	*P* value
Any antibiotic exposure, *n* (%)	53 (76.8%)	29 (82.9%)	0.201
Antibiotic exposure pattern, *n* (%)			<0.001
Prolonged or repeated exposure	16 (23.2%)	28 (80%)	
Standard course	29 (42.0%)	3 (8.6%)	
Single short course	8 (11.6%)	0	
None	16 (23.2%)	4 (11.4%)	
Specific antibiotic classes used, *n* (%)			
Macrolides	43 (62.3%)	30 (85.7%)	0.017
Cephalosporins/β-lactams	25 (36.2%)	18 (51.4%)	0.137
Tetracyclines	2 (2.9%)	12 (34.3%)	<0.001
Sulfonamides	3 (4.3%)	1 (2.9%)	1.000
Quinolones	0	2 (5.7%)	0.111
Concomitant medications, *n* (%)			
Antiviral drugs	7 (10.1%)	6 (17.1%)	0.308
Systemic corticosteroids	29 (42.0%)	18 (51.4%)	0.362

### *In vitro* and *in silico* capsule typing

The *bexA* gene is involved in capsule expression in Hi, which contributes to bacterial immune evasion and pathogenicity. Polymerase chain reaction (PCR) amplification of the *bexA* gene revealed that out of 104 isolates, only one strain yielded a 343 bp amplicon, indicating a typeable strain, while the remaining 103 isolates yielded no amplicon (Fig. S1). Furthermore, *in silico* capsule typing using the Hicap tool identified a complete serotype f-specific capsule locus exclusively in the single *bexA*-positive isolate. To confirm this Hif isolate, the phenotypic and genomic features of this solitary isolate were additionally characterized using transmission electron microscopy and circular genome mapping (Fig. S2 and S3).

### Core-genome phylogeny and MLST profiling

**Fig 1:**
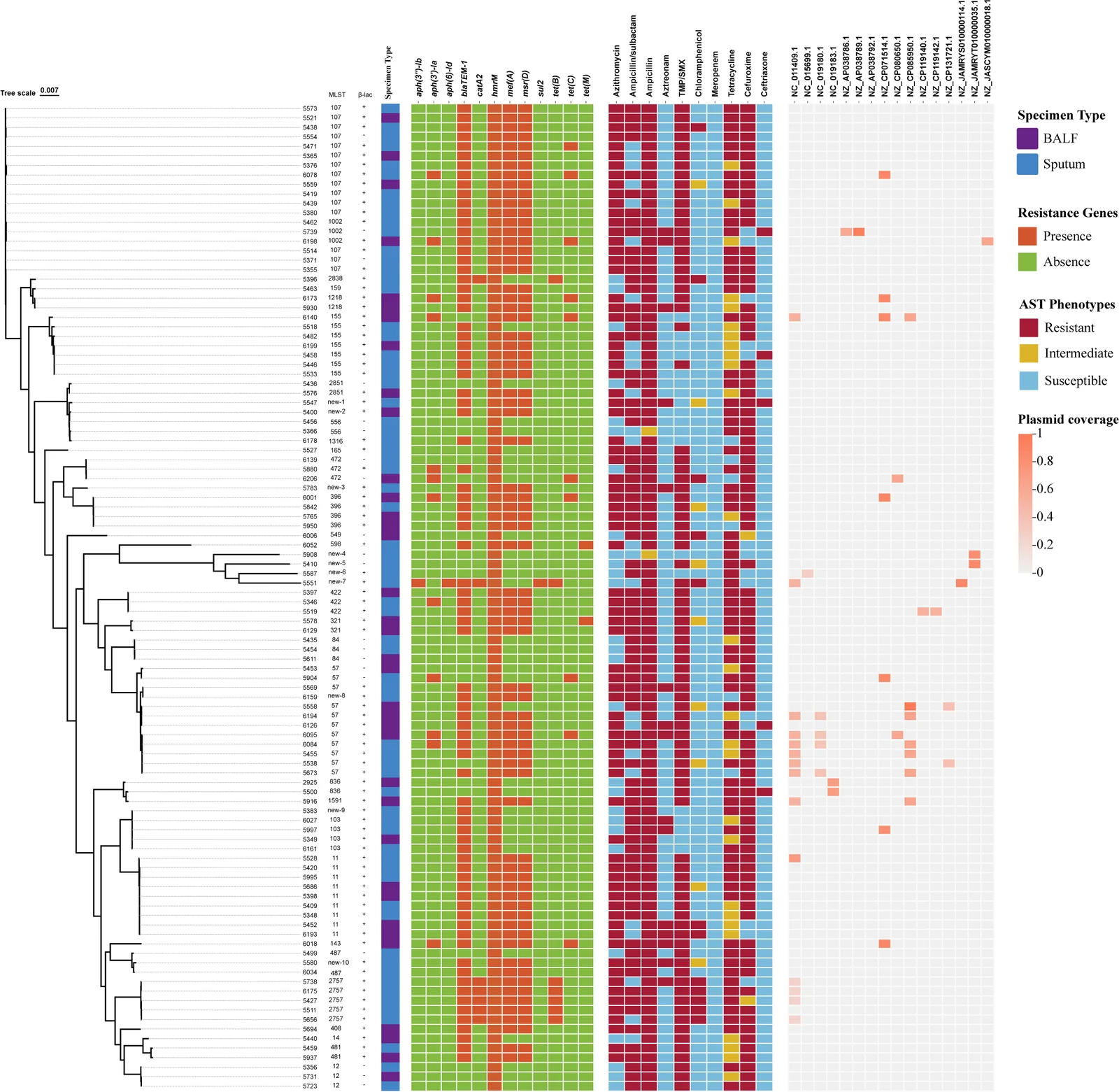
Comprehensive core-genome-based phylogenetic tree and integrated profiles of the multidrug-resistant *Haemophilus influenzae* isolates. The phylogenetic tree on the left was constructed based on core-genome alignments. The adjacent annotations, from left to right, display the sequence type (ST), specimen type, β-lactamase production phenotype (β-lac), antimicrobial resistance gene carriage, phenotypic AST results, and plasmid coverage for each individual isolate. Specimen type: purple indicates BALF, and blue indicates sputum. β-lac indicates the β-lactamase production status (+ for positive, − for negative). Resistance genes: orange represents the presence of specific resistance determinants, whereas green indicates their absence. AST phenotypes: red, yellow, and light blue denote resistant (R), intermediate (I), and susceptible (S) phenotypes, respectively. Plasmid coverage: the carriage of plasmid replicons is depicted as a continuous heatmap gradient ranging from 0 (absence) to 1 (full coverage).

Following high-quality sequencing and genome assembly, the genome qualities of all 104 isolates were comprehensively evaluated. Detailed sequencing and assembly metrics for each isolate, including sequencing depth, genome size, contig number, and N50, are summarized in Table S2. Furthermore, pan-genome analysis identified a total of 5,729 genes within the study cohort. This pan-genome comprises a core genome of 793 genes (present in ≥99% of the isolates), along with a highly polymorphic accessory genome consisting of soft core (472 genes), shell (996 genes), and cloud (3,468 genes) genomes. To establish a high-resolution evolutionary framework, a comprehensive core-genome phylogenetic tree was constructed (Fig. 1). This phylogeny explicitly visualizes the core-genome evolutionary trajectories alongside the dynamic acquisition of resistance determinants and plasmid replicons.

**Fig 2:**
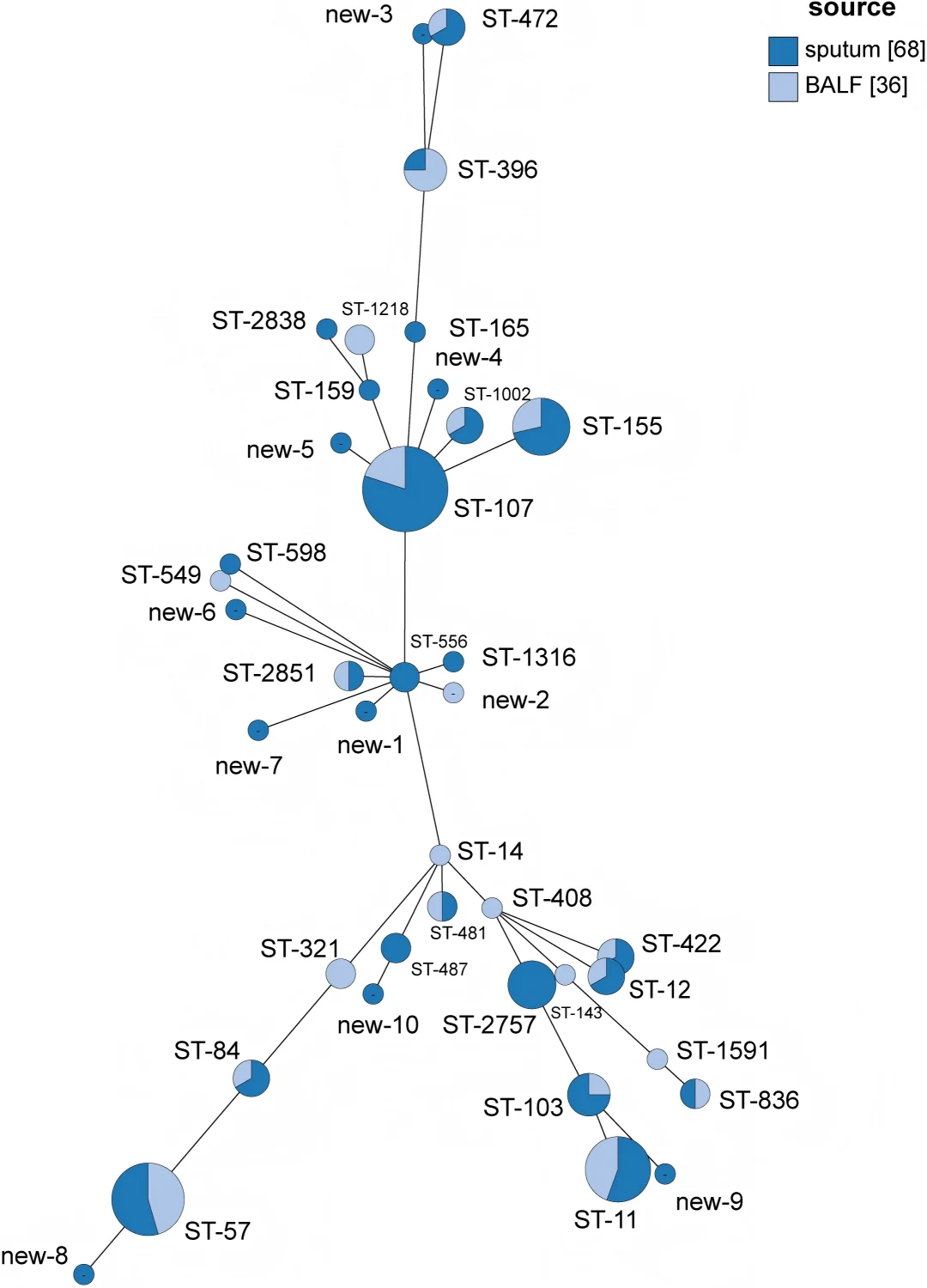
MST of *Haemophilus influenzae* isolates based on MLST. The genetic relationships among the 104 Hi isolates were analyzed based on the allelic profiles of seven standard housekeeping genes. Each circle corresponds to a unique ST, with the circle area being directly proportional to the number of isolates. Colors within the circles indicate the clinical sample source: light blue for BALF and dark blue for sputum. Numbers inside the nodes denote the specific STs, including the 29 known STs identified in this study. Solid lines connecting the nodes indicate single-locus variants (SLVs, representing STs that differ at exactly one housekeeping gene) and double-locus variants (DLVs, representing STs that differ at exactly two housekeeping genes).

Moreover, *in silico* MLST analysis identified 29 distinct known sequence types (STs) and 10 novel STs, and their genetic relationships were evaluated using a Minimum Spanning Tree (MST) (Fig. 2). These 10 novel STs corresponded to 10 sporadic singleton isolates (new-1 to new-10). Notably, both the MST and core-pan genome clustering revealed close phylogenetic relatedness between these novel lineages and major circulating clones. For instance, the isolate belonging to the new-10 sequence type clustered tightly within the ST-57 clade, suggesting that it represents a recent evolutionary derivative of this predominant clone. It was found that ST-107 was the most prevalent (*n* = 15, 14.4%), followed by ST-57 (*n* = 11, 10.6%), ST-11 (*n* = 9, 8.7%), ST-155 (*n* = 7, 6.7%), and ST-2757 (*n* = 5, 4.8%). Although the broad core-genome phylogenetic clustering was generally concordant with the MLST assignments, the WGS-based phylogeny provided a substantially higher discriminatory resolution. For instance, it successfully differentiated isolates within identical STs into distinct sub-clades, demonstrating that while MLST captures the broad evolutionary framework, core-genome phylogeny coupled with the accessory genome exhibits deeper intra-lineage diversity and dynamic genetic alterations.

### Phenotypic antimicrobial resistance profiles

As shown in the results of antimicrobial susceptibility testing (Table 2), the highest resistance rate of 104 Hi isolates was observed for ampicillin (98.1%, 102/104), followed by cefuroxime (84.6%, 88/104), trimethoprim/sulfamethoxazole (81.7%, 85/104), ampicillin–sulbactam (76.0%, 79/104), azithromycin (74.0%, 77/104), and tetracycline (69.2%, 72/104). The highest susceptibility was to meropenem (100%, 104/104), followed by ceftriaxone (95.2%, 99/104), aztreonam (85.6%, 89/104), and chloramphenicol (79.8%, 83/104). High resistance rates exceeding 70% were observed for multiple antibiotics in both groups. In the BALF group (*n* = 35), these included ampicillin (100%, 35/35), trimethoprim/sulfamethoxazole (85.7%, 30/35), azithromycin (82.9%, 29/35), and cefuroxime (77.1%, 27/35). All isolates from BALF were susceptible to meropenem (100%, 35/35), with susceptibility rates of 97.1% (34/35) for ceftriaxone and 77.1% (27/35) for chloramphenicol. In the sputum group (*n* = 69), high resistance was similarly recorded for ampicillin (97.1%, 67/69), cefuroxime (88.4%, 61/69), trimethoprim/sulfamethoxazole (79.7%, 55/69), ampicillin–sulbactam (79.7%, 55/69), and tetracycline (79.7%, 55/69), while susceptibility to meropenem, ceftriaxone, and chloramphenicol was 100% (69/69), 94.2% (65/69), and 81.2% (56/69), respectively. Overall, Hi exhibited high resistance to β-lactam antibiotics, particularly ampicillin and cefuroxime, whereas the carbapenem meropenem remained fully active against all isolates. Between the two specimen groups, resistance patterns were not uniformly higher in one group: BALF isolates showed higher resistance to azithromycin and trimethoprim/sulfamethoxazole, whereas sputum isolates showed higher resistance to cefuroxime, ampicillin–sulbactam, and tetracycline.

**TABLE 2:** Antimicrobial resistance and susceptibility profiles of 104 *Haemophilus influenzae* isolates from BALF and sputum samples

	BALF			Sputum			Sum		
	Resistant	Intermediate	Susceptible	Resistant	Intermediate	Susceptible	Resistant	Intermediate	Susceptible
Azithromycin	29 (82.9%)	0	6 (16.7%)	48 (69.6%)	0	21 (30.4%)	77 (74%)	0	27 (26%)
Amp/Sulbactam	24 (68.6%)	0	12 (33.3%)	55 (79.7%)	0	14 (20.3%)	79 (76%)	0	25 (24%)
Ampicillin	35 (100%)	0	0	67 (97.1%)	2 (2.9%)	0	102 (98.1%)	2 (1.9%)	0
Aztreonam	7 (20%)	0	28 (80%)	8 (11.6%)	0	61 (88.4%)	15 (14.4%)	0	89 (85.6%)
Trimeth/Sulfa	30 (85.7%)	0	5 (14.3%)	55 (79.7%)	0	14 (20.3%)	85 (81.7%)	0	19 (18.3%)
Chloramphenicol	4 (11.4%)	4 (11.4%)	27 (77.1%)	8 (11.6%)	5 (7.2%)	56 (81.2%)	12 (11.5%)	9 (8.7%)	83 (79.8%)
Meropenem	0	0	35 (100%)	0	0	69 (100%)	0	0	104 (100%)
Tetracycline	20 (57.1%)	14 (40%)	2 (5.7%)	55 (79.7%)	14 (20.3%)	0	72 (69.2%)	27 (26%)	6 (5.8%)
Cefuroxime	27 (77.1%)	1 (2.9%)	7 (20%)	61 (88.4%)	1 (1.5%)	7 (10.1%)	88 (84.6%)	2 (1.9%)	14 (13.5%)
Ceftriaxone	1 (2.9%)	0	35 (97.1%)	4 (5.8%)	0	65 (94.2%)	5 (4.8%)	0	99 (95.2%)

### Genotypic resistance determinants and plasmid replicons

Based on the Comprehensive Antibiotic Resistance Database (CARD) classification, a total of 12 antibiotic resistance genes were identified across the 104 Hi isolates (Fig. 1). Notably, *hmrM*, which encodes a MATE family multidrug efflux pump, was ubiquitous and detected in all isolates (100%, 104/104). *blaTEM-1* was also highly prevalent (77.9%, 81/104), which is consistent with the overall high level of phenotypic β-lactam resistance observed in this study.

Through genomic analysis of the sequence assemblies, 15 distinct plasmid replicons were identified across 25 isolates. As detailed in the plasmid profiles (Table S3), the annotation of these plasmid-associated contigs revealed the carriage of key antibiotic resistance genes, including *blaTEM-1* (β-lactam resistance), *tet(B)* (tetracycline resistance), cat (chloramphenicol resistance), and *aph(3*′*)-Ia* (aminoglycoside resistance). Notably, the carriage of these plasmid-associated contigs strongly correlated with the actual resistance detection, particularly matching the presence of *blaTEM-1* and *aph(3*′*)-Ia*. The concurrent carriage of these plasmid-associated sequences and multiple resistance genes collectively drives the extensive MDR observed in these isolates.

### Resistance typing of β-lactamase

Antimicrobial susceptibility testing revealed that among the 104 Hi isolates, 102 were resistant and 2 were intermediate to ampicillin (Table 2). Phenotypic screening using the nitrocefin assay identified 84 (80.8%) isolates as β-lactamase-positive ampicillin-resistant (BLPAR) and 20 (19.2%) as β-lactamase-negative ampicillin-resistant (BLNAR) (Fig. 1). To further elucidate the underlying resistance drivers, we performed genotypic stratification based on *blaTEM-1* carriage and *ftsI* mutations (Fig. S4). The genotypic β-lactamase-positive ampicillin-resistant (gBLPAR) class, defined by the presence of the *blaTEM-1* gene without *ftsI* mutations, was the predominant resistance type (*n* = 71, 68.3%). The next most common classes were the genotypic β-lactamase-positive amoxicillin-clavulanate-resistant (gBLPACR) class (*blaTEM-1* positive with *ftsI* mutations; *n* = 10, 9.6%) and the genotypic β-lactamase-negative ampicillin-resistant (gBLNAR) class (*blaTEM-1* negative with *ftsI* mutations; *n* = 6, 5.8%). Notably, the remaining 17 isolates (16.3%) lacked both the *blaTEM-1* gene and *ftsI* mutations while still exhibiting non-susceptibility to ampicillin. Despite the observed heterogeneity between phenotypic and genotypic profiles, these results indicate that alongside the predominant *blaTEM-1*-driven enzymatic mechanism and target-site modifications, alternative molecular mechanisms may also contribute to ampicillin resistance in this study.

### Virulence gene profiles of NTHi stratified by sequence type and respiratory tract origin

**Fig 3:**
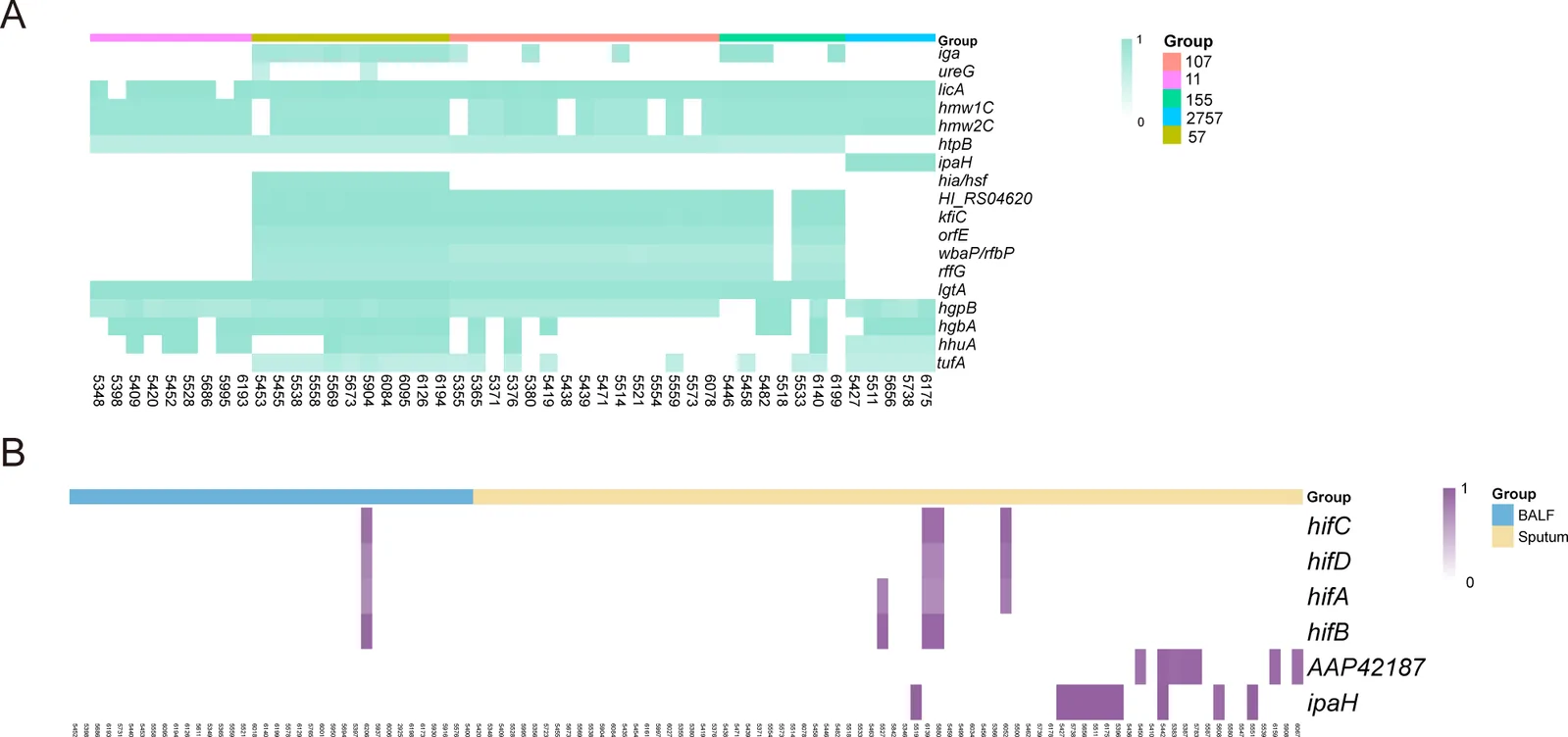
Comparative virulence gene profiles across sequence types and isolation sites. (**A**) Virulence gene profiles of *Haemophilus influenzae* ST-107, ST-57, ST-11, and ST-2757. Distribution of virulence genes in Hi isolates belonging to ST-107 (green, *n* = 15), ST-57 (purple, *n* = 11), ST-11 (red, *n* = 9), and ST-2757 (blue, *n* = 5). Distinct sequence type-associated differences were observed, with ST-107 carrying *iga1*, *hap*, *HI_RS04620*, *kfiC*, *orfE*, *wbaP/rfbP*, *rffG*, and *tufA* that were absent in ST-11, whereas *licD*, *tbpB*, *hgbA*, and *hhuA* were detected in ST-11 but not in ST-107. ST-57 showed ST-specific detection of *hia/hsf* and *iga*, while *ipaH* was detected only in ST-2757. (**B**) Virulence gene profiles of *Haemophilus influenzae* isolates from the upper and lower respiratory tracts. Comparison of virulence gene carriage between Hi isolates from the upper respiratory tract (sputum, pink) and the lower respiratory tract (BALF, blue). *IpaH* was detected exclusively in the sputum group. Capsule-associated genes *bexB*, *bexC*, *bexD*, *hcsA*, *hcsB*, *bexA,* and *hscA* were detected only in the Hif isolate. The 0 to 1 gradient coloring scale represents the normalized sequence identity score, where 1 indicates 100% presence/identity of the gene, and 0 indicates its absence.

Through comparison with the Virulence Factor Database (VFDB), a total of 162 genes associated with virulence and colonization were identified across the 104 Hi isolates (Data set S1). Detailed information on these primary virulence genes, including their encoded protein products and specific biological roles in pathogenesis, is systematically summarized in Table 3. To investigate the genetic characteristics of distinct clonal lineages, our comparative analysis focused on the prevalent sequence types (ST-107, ST-57, ST-11, ST-155, and ST-2757), revealing 18 genes with differential distribution across these dominant lineages (Fig. 3A). The results demonstrated that a gene cluster comprising *HI_RS04620*, *kfiC*, *orfE*, *wbaP/rfbP*, and *rffG*, which is primarily involved in surface glycan assembly and lipooligosaccharide (LOS) synthesis, was widely detected in ST-107, ST-57, and the vast majority of ST-155 isolates, but was completely absent in ST-11 and ST-2757 isolates. Meanwhile, genes associated with iron acquisition, such as *hgbA* and *hhuA*, were detected in a subset of ST-11 strains, but were absent in the ST-107 and ST-155 strains. Furthermore, certain lineages harbored highly lineage-specific marker genes; for instance, the adhesion-related *hia/hsf* gene was almost exclusively identified in ST-57 isolates, whereas the *ipaH* gene was uniquely restricted to ST-2757 isolates. Additionally, we conducted a comparative analysis of the gene carriage profiles between BALF and sputum isolates (Fig. 3B). The results indicated that the invasion-related gene *ipaH* and the adhesion-associated pilus gene cluster (such as *hifA*, *hifB*, *hifC*, and *hifD*) were detected almost exclusively in sputum-derived isolates, but were rarely observed in the BALF group. These findings suggest that the differential distribution of distinct adhesion and invasion factors may reflect the specialized colonization and adaptation strategies of the strains to the local microenvironment of different clinical specimens.

**TABLE 3:** Summary of identified virulence genes in *Haemophilus influenzae* isolates and their roles in pathogenesis

Gene symbol	Protein product	Role in virulence (pathogenic function)
*hap*	*Haemophilus* adhesion and penetration protein	An autotransporter protein that promotes intimate adherence to and penetration of respiratory epithelial cells; also possesses serine protease activity to evade host immunity
*hia*/*hsf*	*H. influenzae* adhesin/*Haemophilus* surface fibril	Major non-pilus adhesins that mediate highly efficient binding and firm attachment to the host respiratory epithelium
*licD*	Phosphorylcholine (ChoP) transferase	Responsible for the addition of ChoP to the lipooligosaccharide (LOS), which mimics host cell structures to evade immunity and promotes binding to the host PAF receptor
*HI_RS04620*	Lipooligosaccharide (LOS) biosynthesis protein	Involved in the biosynthesis and phase variation of LOS, a crucial cell envelope component that mediates endotoxicity, immune evasion, and host cell attachment
*iga*/*iga1*	Immunoglobulin A1 (IgA1) protease	Cleaves human IgA1 antibodies on mucosal surfaces, allowing the bacteria to evade the host’s primary secretory immune defense and facilitate mucosal colonization
*hgbA*/*hhuA*	Hemoglobin/haptoglobin-binding proteins	Receptors that bind host hemoglobin or hemoglobin-haptoglobin complexes, serving as vital mechanisms for scavenging iron during infection
*ipaH*	Invasion plasmid antigen H	Acts as an E3 ubiquitin ligase effector that modulates host cell signaling pathways, potentially facilitating mucosal invasion and dampening the host inflammatory response
*bexA*, *B*, *C*, *D*	Capsule export ATP-binding/transport proteins	Essential components of the ABC transporter system required for the export and assembly of the polysaccharide capsule, a major factor in resisting phagocytosis (specific to encapsulated strains)

## DISCUSSION

NTHi has emerged as a predominant opportunistic pathogen in pediatric respiratory infections (16). In recent years, the escalating prevalence of MDR NTHi strains has posed severe clinical challenges worldwide (2, 14). Understanding the precise genomic architectures underlying this high-level resistance is crucial for effective epidemiological surveillance and therapeutic decision-making (6). In this study, we utilized high-resolution WGS to comprehensively characterize 103 MDR NTHi isolates and one encapsulated isolate. By constructing a comprehensive core-genome-based phylogeny, we established a unified framework that aligns STs, diverse antimicrobial resistance determinants, plasmid replicon profiles, and phenotypic resistance patterns.

MLST has long been used as a standard tool for characterizing the population structure of NTHi. In our study, we identified 39 distinct STs, with ST-107, ST-11, ST-155, ST-2757, and ST-57 being the major circulating lineages. The high proportion of ST-107 aligns with previous reports of its regional spread in China (9–11). Furthermore, consistent with the known limitations of MLST (6, 17), our core-genome phylogenetic analysis provided a more granular view of the genetic variation within these identical STs. By clustering these prevalent STs into distinct sub-clades, the WGS-based framework offered improved resolution for differentiating closely related isolates. This highlights the practical utility of WGS in complementing traditional MLST to better characterize the local population structure and resistance patterns of NTHi.

A significant finding of this study is the high concordance between the predicted resistant genes and phenotypic profiles of AST results. For instance, the widespread phenotypic resistance to β-lactamase perfectly corresponds with the high carriage rate of the *blaTEM-1* gene; similarly, non-susceptibility to macrolides in certain strains is strictly accompanied by the detection of the *mef(A)* and *msr(D)* genes. Additionally, the ubiquitous presence of the efflux pump gene *hmrM* provides an intrinsic background for MDR. More importantly, our integrated analysis reveals the pivotal role of horizontal gene transfer in shaping these MDR strains. As evidenced by our plasmid replicon heatmap, specific plasmids frequently co-occur with MDR determinants, such as *catA2*, *sul2*, and *tet* variants. This aligns with recent findings that mobile genetic elements, including novel transposons, are primary drivers of high-level MDR in NTHi (18).

Supplementing the plasmid-mediated resistance, chromosomal *ftsI* mutations further bolster β-lactam resistance. In our cohort, alongside the predominant *blaTEM-1*-driven isolates, genomic stratification identified specific *ftsI* mutations in gBLPACR (*n* = 10) and gBLNAR (*n* = 6) isolates. Interestingly, while recent studies have indicated a significant rise in the proportion of BLNAR strains within certain Chinese pediatric cohorts (19), the prevalence of the gBLNAR genotype in this study remains relatively low (5.8%). This suggests that *ftsI*-driven resistance is not currently the primary reason. Nevertheless, these specific amino acid substitutions in PBP3, as highlighted by Zhang et al. and Wan et al. (14, 18), significantly reduce susceptibility to various cephems. Furthermore, the synergy between β-lactamase production and *ftsI* target-site mutations observed in our gBLPAR isolates creates a formidable defense line against β-lactam therapy, warranting continuous clinical surveillance.

Beyond antimicrobial resistance, our analysis observed that different STs exhibited lineage-specific virulence profiles. This finding aligns with early pioneering studies that highlighted the antigenic diversity of virulence factors (20, 21), as well as with the well-established Hi supragenome model (6, 17). This diversity is further exemplified by the specific allelic variation of *IgA* proteases (e.g., *igaA1*, *igaA2*) expressed during respiratory adaptation (22). Coupled with our clinical metadata, these findings suggest that the coexistence of these specific virulence backgrounds with high-level MDR phenotypes likely reflects the dual selective pressures of micro-niche adaptation and rigorous clinical antibiotic screening. Such selective pressures may drive the local enrichment and persistence of specific resistant lineages with inherent environmental adaptability.

Despite providing genomic insights, this study has certain limitations. First, its regional retrospective design limits geographical coverage, necessitating future multicenter longitudinal validation. Second, because we relied on short-read sequencing, we could not definitively determine whether the identified plasmid-associated sequences existed as extrachromosomal episomes or were integrated into the chromosome. Furthermore, the functions of these lineage-specific virulence factors are inferred primarily from *in silico* genomic analyses. The precise contributions of these factors to *in vivo* pathogenicity require further functional validation via transcriptomic studies or animal models. In conclusion, our findings demonstrate that under intense clinical and environmental selective pressures, MDR NTHi populations exhibit a strong coupling between specific distinct evolutionary lineages and acquired resistance determinants. These findings highlight the persistent threat posed by locally adapted, high-risk lineages and underscore the need for continuous high-resolution genomic surveillance to inform antibiotic stewardship and infection control strategies.

## MATERIALS AND METHODS

### Strain sources

A total of 104 MDR Hi strains were intentionally selected and included in this study. These strains were isolated from respiratory samples, including sputum and BALF, from hospitalized pediatric patients at the Children’s Hospital of the Capital Institute of Pediatrics between January 2024 and January 2025. This study has been approved by the Medical Ethics Committee of the Capital Institute of Pediatrics (approval no. SHERLLM2024051).

### Isolation and bacterial identification

The clinical respiratory samples were inoculated onto chocolate agar plates supplemented with vancomycin (Guangzhou Dijing Biotechnology Co., Ltd., Guangzhou, China) to selectively isolate Hi. The plates were incubated at 35°C in a 5% CO₂ atmosphere for 18–24 h using an MCO-5AC CO₂ incubator (Sanyo, Japan). Suspected colonies were subsequently identified to the species level using MALDI-TOF MS (bioMérieux, France).

### Antimicrobial susceptibility testing and phenotypic screening

AST of Hi isolates was performed using the VITEK 2 Compact system (bioMérieux, France; software version 9.04.4), following the manufacturer’s instructions. Bacterial suspensions were prepared from fresh cultures adjusted to a 0.5 McFarland standard and inoculated into AST cards according to the guidelines provided by bioMérieux. The cards were automatically filled, sealed, and incubated in the instrument. The minimum inhibitory concentrations (MICs) obtained from the VITEK 2 system were automatically read and analyzed by the Advanced Expert System (AES; version 9.02). The MIC values were interpreted according to the clinical breakpoints outlined in the Clinical and Laboratory Standards Institute Supplement M100, 34th edition (2024) (23). Furthermore, the phenotypic detection of β-lactamase production was performed using the nitrocefin disk colorimetric method (Biobw, Beijing, China). Briefly, the bacterial isolates were applied to moistened nitrocefin disks, and a positive reaction was defined as a color change from yellow to red within 10 min. Staphylococcus aureus ATCC 29213 was utilized as a positive quality control in each batch of experiments.

### DNA extraction, whole-genome sequencing, and assembly

Bacterial genomic DNA was extracted using the TIANamp Bacteria Genomic DNA Kit (TianGen Biotech Co., Ltd., China). The extracted DNA was washed with an ethanol-based buffer, eluted in nuclease-free water, and stored at –80°C for future use. The genomic DNA was then sent to Shanghai Meiji Biological Pharmaceutical Technology Co., Ltd., where whole-genome sequencing was performed using the Illumina X Plus sequencing platform. The raw sequencing data underwent quality control using FastQC (version 0.11.2), and genome assembly was completed using SPAdes software (version 3.15.3) (24).

### Capsule genotyping and multi-locus sequence typing

The genomic DNA of Hi strains was used as a template for PCR amplification of the *bexA* gene to determine whether the strain was encapsulated or non-encapsulated. The primer sequences for the bexA gene were adopted from a previous study (25); the upstream primer sequence for the *bexA* gene was 5′-CGTTTGTATGATGTTGATCCA-3′, and the downstream primer sequence was 5′-TGTCCATGTCTTCAAAATG-3′. The PCR amplification conditions were as follows: an initial denaturation at 94°C for 4 min, followed by 35 cycles consisting of denaturation at 94°C for 30 s, annealing at 54°C for 30 s, and extension at 72°C for 45 s; a final extension at 72°C for 5 min. The PCR product was 343 bp, and gel electrophoresis was performed to analyze the presence of the *bexA* gene in the sample (Fig. S1). Determination of the strain serotype was performed using Hicap software (version 1.0.3, https://github.com/scwatts/hicap) (26) to analyze the assembled whole-genome sequencing data, identify capsule biosynthesis loci, and annotate serotypes. MLST was conducted using the MLST software (version 2.19.0, https://github.com/tseemann/mlst). Furthermore, to visualize the genetic relationships among the isolates, a Minimum Spanning Tree (MST) based on the allelic profiles of the seven standard MLST housekeeping genes was constructed using the GrapeTree tool (10) (implementing the MST V2 algorithm) integrated within the PubMLST database platform (https://pubmlst.org/) (27).

### Core-genome phylogenetic analysis

To elucidate the evolutionary dynamics and the core/pan-genome architecture of the isolates, the assembled genomes were processed using Roary software (version 3.13.0, https://sanger-pathogens.github.io/Roary/) to extract both the core-genome alignment and the pan-genome presence/absence matrix. A maximum-likelihood phylogenetic tree was subsequently constructed based on the concatenated core-genome alignment using FastTree (version 2.1.11, http://www.microbesonline.org/fasttree/). The resulting high-resolution core-pan phylogenetic tree was visualized and appropriately rooted using the Interactive Tree Of Life web tool (iTOL, https://itol.embl.de/).

### Genomic profiling of resistome, virulome, and plasmids

To characterize the functional genomic features, the ABRicate software (version 1.0.1, https://github.com/tseemann/abricate) was utilized to screen the whole-genome sequences for antibiotic resistance genes and virulence genes. Specifically, antimicrobial resistance genes were identified using the CARD, and virulence factors were annotated by comparing the sequences against the VFDB (http://www.mgc.ac.cn/VFs/) (28). Furthermore, to investigate the presence of mobile genetic elements, plasmid replicons and associated contigs were identified and characterized using PlasmidFinder (https://cge.food.dtu.dk/services/PlasmidFinder/). The distribution profiles of these genetic elements and metadata were then systematically integrated into the aforementioned phylogenetic tree. All comparative analysis plots were constructed and visually represented using R software (version 4.4.0, https://www.R-project.org/) (28).

### Identification of *ftsI* mutations and BLNAR profiling

To elucidate the molecular basis of ampicillin resistance, ampicillin-non-susceptible isolates were phenotypically and genotypically profiled. Phenotypic β-lactamase production was determined using the nitrocefin disk test. For genotypic stratification, the presence of the *blaTEM-1* gene was determined from the assembled genomes as part of the resistome analysis. To identify mutations in the *ftsI* gene, the full-length *ftsI* sequences were extracted from the genome assemblies using BLASTn (version 2.13.0, https://blast.ncbi.nlm.nih.gov/Blast.cgi). The extracted nucleotide sequences were translated into amino acid sequences and aligned against the reference *PBP3* sequence of Hi strain Rd KW20 (GenBank accession number NC_000907) to identify specific amino acid substitutions associated with resistance.

### Statistical analysis

All statistical analyses were performed using SPSS Statistics software (version 26.0, IBM Corp., USA). Categorical variables, including antimicrobial resistance rates and the prevalence of specific genetic elements across different lineages, were expressed as frequencies and percentages. Comparisons of categorical data between groups were evaluated using the Chi-square test or Fisher’s exact test, as appropriate. A two-sided *P* value of <0.05 was considered to indicate statistical significance.

## Data Availability

The whole-genome sequencing data generated in this study have been deposited in the Genome Sequence Archive (GSA) at the National Genomics Data Center (NGDC), China National Center for Bioinformation (CNCB), and are publicly accessible under BioProject accession number PRJCA049810. A comprehensive metadata table aligning the specific strain numbers used throughout this manuscript with their corresponding CNCB BioSample accessions and clinical characteristics is provided in Table S1.

## SUPPLEMENTAL MATERIAL

Data set S1 spectrum.00498-26-s0001.xlsx Comprehensive virulence gene profiles of 104 *Haemophilus influenzae* isolates. This spreadsheet details the presence/absence matrix and sequence identity scores of 162 virulence factors annotated using the Virulence Factor Database (VFDB) across all 104 sequenced isolates in this study.

File S1 spectrum.00498-26-s0002.txt The Newick string format of the genome-scale core-genome phylogenetic tree for the 104 *Haemophilus* influenzae isolates.

Supplemental material spectrum.00498-26-s0003.docx Supplemental methods, figures, tables, and file providing methodological details, TEM capsule imaging, PCR validation, PBP3 mutation profiles, and comprehensive genomic assembly and plasmid data for the 104 *Haemophilus influenzae* isolates investigated in this study.

ASM does not own the copyrights to Supplemental Material that may be linked to, or accessed through, an article. The authors have granted ASM a non-exclusive, world-wide license to publish the Supplemental Material files. Please contact the corresponding author directly for reuse.
