# Supplementary material for: Multidrug resistance and genomic characteristics of nontypeable Haemophilus influenzae isolates from the respiratory tract of pediatric patients
Source: Microbiol Spectr. 2026 Aug 14;14(9):e00498-26. doi: 10.1128/spectrum.00498-26 (PMC13532297; doi:10.1128/spectrum.00498-26)
Supplement: Supplementary file 1 [file spectrum.00498-26-s0003.docx]

**Supplementary material**

**Methods**

*Transmission Electron Microscopy*

Two Hi strains, one encapsulated (Hif) and one non-encapsulated (NTHi), were examined by TEM using a negative staining method. Bacteria were cultured overnight on chocolate agar plates at 37 °C with 5% CO₂, harvested, and resuspended in PBS. A 5–10 µL suspension was placed on carbon-coated copper grids (Zhongjingyi Technology Co., Ltd., China) and allowed to adsorb for 1–2 min. Excess liquid was removed by gently touching the grid with filter paper. The grids were then stained with 2% phosphotungstic acid (pH ≈ 7.0) (Solarbio Life Sciences, Beijing, China) for 30–60 s, blotted again, and air-dried. Samples were observed using a transmission electron microscope operated at 80 kV, and images were captured at ×500 magnification. (Supplementary figure 2)

**Supplementary Figure**

**Supplementary figure 1** Representative gel electrophoresis of *bexA* PCR amplification. Marker: DNA molecular weight marker; NTHi: Representative non-typeable isolates lacking the *bexA* target band; Hif: The single serotype f isolate showing the expected 343 bp positive band (indicating a typeable, encapsulated strain); NC: Negative control. The presence of the 343 bp amplicon is expected for a typeable strain, while its absence confirms a non-encapsulated (NTHi) strain.

**Supplementary figure 2 Capsule visualisation by TEM.** (A) *Haemophilus influenzae*  serotype f. (Hin 6025); (B) Nontypeable *Haemophilus influenzae* (Hin-6175)

**Supplementary figure 3 Genome sequencing analysis of the Hif strain**

(A) Circular diagram of the genome of Hif using CGView Comparison Tool. From outside to inside: circle 1, COG functional categories for forward strand coding sequences, and the different colors represent different COG categories; circle 2, Forward strand sequence features; circle 3, Reverse strand sequence features; circle 4, COG functional categories for reverse strand coding sequence; circle 5, GC content; circle 6, GC skew.

(B) Chromosome DNA analysis of Hif strain.

**Supplementary Figure 4 Amino acid substitution profiles of *PBP3* (encoded by the *ftsI* gene) in *Haemophilus influenzae* isolates**

The alignment map illustrates the specific amino acid mutations in *penicillin-binding protein 3* (*PBP3*) across the sequenced isolates compared to the reference strain Rd KW20 (bottom row). The y-axis represents individual clinical isolates, while the x-axis indicates the relative amino acid positions along the sequence. Colored boxes with single-letter codes highlight the specific substituted amino acids that differ from the reference sequence. The blank/light gray regions indicate conservation with the reference sequence.

**Supplementary Table**

**Supplementary Table 1 Demographic, clinical characteristics, and CNCB database accessions of the 104 *Haemophilus influenzae* isolates.**

| **Strain number** | **CNCB**  **BioSample Accession** | **Group** | **Gender** | **Age** | **Main Disease** |
| --- | --- | --- | --- | --- | --- |
| 5452  5686  6193  5731  6095  6126  5365  6018  5578  5765  2925  5930  6173  5576  5400  5398  5440  5453  5558  6194  5611  5349  5521  5559  6140  6199  6129  5950  6001  5694  6206  5937  6006  6198  5916  5409  5528  5995  5356  5723  5455  5569  5904  5435  5454  6027  6161  5380  5419  5438  5439  5514  5554  5573  5458  5533  5527  5842  5346  5519  5880  6139  6034  5366  5456  6052  5500  5462  6178  5427  5656  6175  5396  5383  5410  5547  5580  5587  6159  5397  5348  5420  5538  5673  6084  5997  5355  5371  5376  5471  6078  5446  5482  5518  5463  5459  5499  5739  5511  5738  5436  5551  5783  5908 | SAMC6550209:2025021100076_Hin5452  SAMC6550207:2025021300073_Hin5686  SAMC6550206:2025030500002_Hin6193  SAMC6550223:2025021300076_Hin5731  SAMC6550191:2025030500008_Hin6095  SAMC6550185:2025030500025_Hin6126  SAMC6550135:2025021100068_Hin5365  SAMC6550215:2025030500016_Hin6018  SAMC6550178:2025021300096_Hin5578  SAMC6550155:2025021300082_Hin5765  SAMC6550195:2025021300074_Hin2925  SAMC6550145:2025030500027_Hin5930  SAMC6550144:2025030500010_Hin6173  SAMC6550169:2025021300091_Hin5576  SAMC6550171:2025021100052_Hin5400  SAMC6550208:2025021100077_Hin5398  SAMC6550225:2025021100060_Hin5440  SAMC6550183:2025021100067_Hin5453  SAMC6550186:2025021300085_Hin5558  SAMC6550187:2025030500018_Hin6194  SAMC6550182:2025021300094_Hin5611  SAMC6550202:2025021100070_Hin5349  SAMC6550136:2025021300132_Hin5521  SAMC6550125:2025021300087_Hin5559  SAMC6550146:2025030500003_Hin6140  SAMC6550150:2025030500014_Hin6199  SAMC6550179:2025030500013_Hin6129  SAMC6550157:2025030500030_Hin5950  SAMC6550154:2025030500001_Hin6001  SAMC6550221:2025021300083_Hin5694  SAMC6550161:2025030500017_Hin6206  SAMC6550227:2025030500026_Hin5937  SAMC6550174:2025030500029_Hin6006  SAMC6550129:2025030500028_Hin6198  SAMC6550197:2025030500023_Hin5916  SAMC6550211:2025021100083_Hin5409  SAMC6550205:2025021300125_Hin5528  SAMC6550204:2025030500009_Hin5995  SAMC6550222:2025021100065_Hin5356  SAMC6550224:2025021300079_Hin5723  SAMC6550192:2025021100072_Hin5455  SAMC6550188:2025021300095_Hin5569  SAMC6550184:2025030500005_Hin5904  SAMC6550180:2025021100057_Hin5435  SAMC6550181:2025021100079_Hin5454  SAMC6550199:2025030500020_Hin6027  SAMC6550200:2025030500006_Hin6161  SAMC6550124:2025021100046_Hin5380  SAMC6550130:2025021100063_Hin5419  SAMC6550140:2025021100069_Hin5438  SAMC6550131:2025021100075_Hin5439  SAMC6550137:2025021300124_Hin5514  SAMC6550141:2025021300084_Hin5554  SAMC6550126:2025021300089_Hin5573  SAMC6550151:2025021100042_Hin5458  SAMC6550152:2025021300127_Hin5533  SAMC6550153:2025021300130_Hin5527  SAMC6550156:2025021300075_Hin5842  SAMC6550176:2025021100047_Hin5346  SAMC6550177:2025021300121_Hin5519  SAMC6550159:2025030500011_Hin5880  SAMC6550160:2025030500007_Hin6139  SAMC6550214:2025030500022_Hin6034  SAMC6550167:2025021100078_Hin5366  SAMC6550172:2025021100055_Hin5456  SAMC6550173:2025030500031_Hin6052  SAMC6550196:2025021300128_Hin5500  SAMC6550128:2025021100054_Hin5462  SAMC6550166:2025030500033_Hin6178  SAMC6550219:2025021100074_Hin5427  SAMC6550220:2025021300090_Hin5656  SAMC6550218:2025030500004_Hin6175  SAMC6550142:2025021100062_Hin5396  SAMC6550198:2025021100081_Hin5383  SAMC6550164:2025021100045_Hin5410  SAMC6550170:2025021300123_Hin5547  SAMC6550213:2025021300093_Hin5580  SAMC6550162:2025021300086_Hin5587  SAMC6550189:2025030500012_Hin6159  SAMC6550175:2025021100049_Hin5397  SAMC6550210:2025021100058_Hin5348  SAMC6550203:2025021100056_Hin5420  SAMC6550194:2025021300122_Hin5538  SAMC6550193:2025021300092_Hin5673  SAMC6550190:2025030500021_Hin6084  SAMC6550201:2025030500015_Hin5997  SAMC6550132:2025021100043_Hin5355  SAMC6550133:2025021100080_Hin5371  SAMC6550138:2025021100066_Hin5376  SAMC6550134:2025021100073_Hin5471  SAMC6550139:2025030500019_Hin6078  SAMC6550149:2025021100061_Hin5446  SAMC6550148:2025021100071_Hin5482  SAMC6550147:2025021300126_Hin5518  SAMC6550143:2025021100059_Hin5463  SAMC6550226:2025021100051_Hin5459  SAMC6550212:2025021100048_Hin5499  SAMC6550127:2025021300077_Hin5739  SAMC6550216:2025021300120_Hin5511  SAMC6550217:2025021300081_Hin5738  SAMC6550168:2025021100053_Hin5436  SAMC6550165:2025021300129_Hin5551  SAMC6550158:2025021300080_Hin5783  SAMC6550163:2025030500024_Hin5908 | BALF  BALF  BALF  BALF  BALF  BALF  BALF  BALF  BALF  BALF  BALF  BALF  BALF  BALF  BALF  BALF  BALF  BALF  BALF  BALF  BALF  BALF  BALF  BALF  BALF  BALF  BALF  BALF  BALF  BALF  BALF  BALF  BALF  BALF  BALF  Sputum  Sputum  Sputum  Sputum  Sputum  Sputum  Sputum  Sputum  Sputum  Sputum  Sputum  Sputum  Sputum  Sputum  Sputum  Sputum  Sputum  Sputum  Sputum  Sputum  Sputum  Sputum  Sputum  Sputum  Sputum  Sputum  Sputum  Sputum  Sputum  Sputum  Sputum  Sputum  Sputum  Sputum  Sputum  Sputum  Sputum  Sputum  Sputum  Sputum  Sputum  Sputum  Sputum  Sputum  Sputum  Sputum  Sputum  Sputum  Sputum  Sputum  Sputum  Sputum  Sputum  Sputum  Sputum  Sputum  Sputum  Sputum  Sputum  Sputum  Sputum  Sputum  Sputum  Sputum  Sputum  Sputum  Sputum  Sputum  Sputum | Male  Female  Male  Male  Male  Male  Male  Male  Male  Male  Male  Male  Male  Male  Male  Female  Female  Female  Female  Female  Female  Female  Female  Female  Female  Female  Female  Female  Female  Female  Female  Female  Female  Female  Female  Male  Male  Male  Male  Male  Male  Male  Male  Male  Male  Male  Male  Male  Male  Male  Male  Male  Male  Male  Male  Male  Male  Male  Male  Male  Male  Male  Male  Male  Male  Male  Male  Male  Male  Male  Male  Male  Male  Male  Male  Male  Male  Male  Male  Female  Female  Female  Female  Female  Female  Female  Female  Female  Female  Female  Female  Female  Female  Female  Female  Female  Female  Female  Female  Female  Female  Female  Female  Female | 5  9  5  9  6  8  1  6  10  5  10  6  <1y  1  1  9  1  4  1  6  8  7  5  5  10  3  3  9  8  7  7  4  9  7  8  1  0  9  12  9  1  2  <1y  <1y  <1y  <1y  10  <1y  7  <1y  <1y  1  13  4  <1y  <1y  7  2  <1y  1  <1y  <1y  <1y  <1y  5  <1y  <1y  1  <1y  2  2  8  <1y  <1y  <1y  1  8  <1y  <1y  7  <1y  <1y  <1y  <1y  1  <1y  15  <1y  1  1  <1y  <1y  1  <1y  <1y  1  <1y  6  <1y  8  <1y  10  5  <1y | Mycoplasma  Mixed  Bacterial  Bacterial  Mycoplasma  Viral  Mixed  Mixed  Others  Viral  Mixed  Others  Mixed  Mixed  Mixed  Others  Mixed  Bacterial  Mixed  Mixed  Mixed  Mixed  Others  Mixed  Mixed  Mixed  Bacterial  Mycoplasma  Mixed  Mixed  Mixed  Mycoplasma  Mixed  Mixed  Others  Mixed  Mixed  Others  Mixed  Bacterial  Mixed  Mixed  Viral  Mixed  Others  Mixed  Others  Bacterial  Mixed  Mixed  Mixed  Mixed  Mixed  Mixed  Mixed  Mixed  Bacterial  Mixed  Mixed  Mixed  Viral  Mixed  Mixed  Mixed  Mixed  Mixed  Bacterial  Mixed  Mixed  Mixed  Bacterial  Mycoplasma  Viral  Viral  Mixed  Mixed  Mixed  Mixed  Mixed  Mycoplasma  Mixed  Mixed  Mixed  Viral  Mixed  Mixed  Mixed  Mixed  Mixed  Mixed  Mixed  Mixed  Mixed  Mixed  Bacterial  Mixed  Mixed  Others  Bacterial  Mixed  Mixed  Others  Others  Viral |

In the "Main Disease" column, "Mycoplasma", "Bacterial", and "Viral" refer to Mycoplasma pneumonia, bacterial pneumonia, and viral pneumonia, respectively. BALF: bronchoalveolar lavage fluid; CNCB: China National Center for Bioinformation.

**Supplementary Table 2 Summary of sequencing data quality and genome assembly metrics for all isolates.**

| **Sample Name** | **Read Len (bp)** | **Clean Q30 (%)** | **Depth** | **Genome Size (bp)** | **Scaffold Count** | **N50 (bp)** | **GC Content (%)** | **Completeness**  **(%)** |
| --- | --- | --- | --- | --- | --- | --- | --- | --- |
| 2925  5346  5348  5349  5355  5356  5365  5366  5371  5376  5380  5383  5396  5397  5398  5400  5409  5410  5419  5420  5427  5435  5436  5438  5439  5440  5446  5452  5453  5454  5455  5456  5458  5459  5462  5463  5471  5482  5499  5500  5511  5514  5518  5519  5521  5527  5528  5533  5538  5547  5551  5554  5558  5559  5569  5573  5576  5578  5580  5587  5611  5656  5673  5686  5694  5723  5731  5738  5739  5765  5783  5842  5880  5904  5908  5916  5930  5937  5950  5995  5997  6001  6006  6018  6027  6034  6052  6078  6084  6095  6126  6129  6139  6140  6159  6161  6173  6175  6178  6193  6194  6198  6199  6206 | 151  151  151  151  151  151  151  151  151  151  151  151  151  151  151  151  151  151  151  151  151  151  151  151  151  151  151  151  151  151  151  151  151  151  151  151  151  151  151  151  151  151  151  151  151  151  151  151  151  151  151  151  151  151  151  151  151  151  151  151  151  151  151  151  151  151  151  151  151  151  151  151  151  151  151  151  151  151  151  151  151  151  151  151  151  151  151  151  151  151  151  151  151  151  151  151  151  151  151  151  151  151  151  151 | 96.8365  97.2757  97.1318  97.3315  96.7287  97.5174  97.4022  97.3061  97.2877  97.3485  97.2975  96.2348  96.179  95.7971  95.9158  94.9907  96.2149  96.1765  95.8179  96.0765  96.6187  95.4444  95.9585  95.7839  96.0719  96.1649  94.9168  96.0378  95.9481  96.1134  95.1445  96.2129  95.8216  95.1917  95.9409  96.4011  96.2677  95.9789  95.9866  96.1147  96.1956  96.1346  96.1172  95.7182  96.1963  96.2671  96.2428  96.1017  96.139  96.1157  96.0813  96.2868  96.1923  96.1816  96.0762  96.279  96.1549  96.266  96.1997  96.0891  96.0895  96.0637  96.1434  96.8295  96.9083  96.8755  96.8725  96.8476  96.8757  96.7462  96.8917  96.8121  97.1634  97.243  97.1867  97.2103  97.212  97.1126  97.2147  97.1926  97.2074  96.9503  97.1565  97.1911  96.2882  97.1264  96.8183  97.1795  97.1667  97.1278  97.1935  97.1821  97.1537  97.1873  97.1951  97.17  97.2267  97.1476  97.0523  97.2304  97.1043  96.9157  97.1563  97.2265 | 639.87  733.29  669.83  700.19  651.33  678.26  752.24  779.64  741.64  731.23  691.27  571.09  563.9  668.89  621.08  601.97  581.22  748.52  618.07  553.14  581.46  553.3  720.05  770.24  759.48  588.08  748.79  711.66  663.63  572.94  604.99  595.45  707.81  687.78  856.44  516.58  780.98  564.34  770.65  582.82  616.76  594.2  561.27  277.77  663.62  609.95  558.4  535.81  640.18  570.66  566.48  594.81  619.36  586.71  539.97  565.98  566.93  548.05  582.86  572.56  536.03  561.54  599.29  675.82  687.21  723.59  564.46  709.19  605.89  588.21  336.48  666.15  658.39  544.51  615.82  661.79  569.63  533.86  538.9  576.92  572.26  597.97  538.41  507.93  666.95  553.81  583.85  594.88  546.62  524.22  503.33  603.08  576.54  624.66  270.32  558.53  589.6  535.35  585.86  576.47  537.71  578.91  535.78  587.11 | 1858531  1975377  1880302  1899020  1837129  1802560  1890748  1769388  1834800  1890760  1840498  1985270  1892452  1969438  1880308  1834047  1878157  1879530  1836861  1916671  1903014  1904580  1772802  1835395  1836529  1833324  1880145  1882021  1878277  1881346  1970572  1770728  1824637  1883853  1840103  1884416  1888214  1909086  1777450  1854601  1865138  1840310  1811070  4063910  1836817  1780528  1919939  1829233  1958906  1931118  1982052  1835244  1946283  1884677  1983089  1836111  1874382  1978653  1836559  1986889  1952568  1865069  1975754  1880589  1875906  1798263  1806851  1864522  1869575  1885266  3808335  1887424  1844077  1895883  1856892  1920414  1886279  1833287  1889749  1879638  1906041  1883691  1870483  1923823  1899298  1840724  1884334  1896457  1976020  1981376  1969095  1946836  1783851  1893290  3977482  1899970  1890081  1906340  1878167  1957813  1972324  1840367  1827154  1791445 | 20  38  27  29  18  17  13  36  29  16  25  95  35  36  26  31  30  19  25  22  25  43  33  19  32  22  33  27  46  36  56  32  41  28  25  36  28  38  22  19  28  25  41  74  25  51  32  28  63  24  29  28  54  31  35  32  43  33  29  39  50  30  55  28  35  34  21  27  30  46  122  46  21  38  15  18  33  19  44  19  26  51  25  21  22  31  15  18  49  47  44  30  29  30  95  24  32  23  32  29  52  31  38  21 | 232600  117520  266209  270734  206606  402736  421251  136926  200165  420150  201002  271424  293654  123086  266285  180932  266280  292350  181080  381861  373078  150612  147854  359457  163590  405605  213494  266900  117747  158087  98000  144062  155165  247635  205983  143147  180662  248181  181327  232485  372262  200876  192082  156632  345191  136977  266060  247483  79992  352706  196935  199838  102114  200152  155830  164693  145039  212894  181848  195544  150551  371580  97983  266843  202946  137148  402716  372254  200821  198679  83626  198974  382621  132533  446535  232866  155126  409822  121787  356573  271180  127851  135912  233044  271200  134134  359601  419896  101982  123436  106579  204610  171080  189035  113270  270069  244079  446992  154351  306365  90393  189779  170500  382520 | 38.03  38.08  37.94  38.03  37.88  37.99  37.95  37.94  37.89  37.92  37.87  38.01  37.97  38.07  37.93  37.92  37.93  38.22  37.87  37.98  38.02  38.03  37.94  37.87  37.88  37.96  37.92  37.94  38.1  37.96  38.17  37.93  37.88  38.01  37.89  37.95  37.94  37.98  37.91  38.01  37.93  37.9  37.94  45.26  37.88  37.87  38.01  37.89  38.17  38.07  38.36  37.89  38.21  37.99  38.19  37.92  38.06  38.14  37.92  38.43  38.14  37.95  38.14  37.94  38.05  38.01  37.99  37.93  37.83  37.95  38.16  37.9  37.91  38.12  38.43  38.02  37.94  37.95  37.91  37.95  38.08  37.91  38  38.02  38.04  37.92  37.95  37.94  38.18  38.19  38.14  38.1  37.89  37.93  38.76  38.03  37.97  37.99  37.98  38.05  38.14  37.88  37.85  37.9 | 99.2  99.2  99.2  99.2  98.4  99.2  98.4  97.6  98.4  98.4  98.4  98.4  99.2  99.2  99.2  98.4  99.2  98.4  97.6  99.2  99.2  99.2  99.2  98.4  98.4  99.2  99.2  99.2  99.2  96.8  99.2  99.2  99.2  99.2  98.4  99.2  98.4  98.4  99.2  99.2  99.2  98.4  99.2  99.2  98.4  97.6  99.2  99.2  98.4  99.2  98.4  98.4  99.2  98.4  99.2  98.4  99.2  99.2  99.2  98.4  99.2  99.2  99.2  99.2  99.2  99.2  99.2  99.2  98.4  98.4  89.5  98.4  99.2  99.2  98.4  99.2  99.2  99.2  99.2  99.2  99.2  99.2  99.2  99.2  99.2  99.2  99.2  98.4  99.2  99.2  99.2  99.2  99.2  99.2  98.4  99.2  99.2  99.2  99.2  99.2  99.2  98.4  99.2  99.2 |

**Table 3 Plasmid profiles and associated antibiotic resistance genes identified in 104 *Haemophilus influenzae* isolates**

| Plasmid code | No. of isolates | Antibiotic-resistant genes | Predicted function |
| --- | --- | --- | --- |
| NC_011409.1 | 14 | tet(B) | Chloramphenicol acetyltransferase (drug inactivation); tetracycline MFS efflux pump (drug export); class A β-lactamase TEM-1 (β-lactam hydrolysis); TetR(B) regulator (frameshifted). |
|  |  | catII |  |
|  |  | blaTEM-1 |  |
| NC_015699.1 | 1 | - | - |
| NC_019180.1 | 4 | blaTEM-1 | Encodes a broad-spectrum class A β-lactamase that hydrolyzes β-lactam antibiotic, conferring β-lactam resistance; plasmid also carries rep and mobV genes indicating self-replication and mobilization potential. |
| NC_019183.1 | 2 | blaTEM-1 | Broad-spectrum class A β-lactamase; confers resistance to ampicillin and related β-lactams. Associated with Rep and MobV, suggesting plasmid stability and potential for horizontal transfer. |
| NZ_AP038786.1 | 1 | - | Contains a complete Type IV secretion/conjugation system (Tra/VirB cluster), suggesting plasmid mobilization potential; no known AMR genes detected. Functions mainly in DNA transfer, replication, and maintenance. |
| NZ_AP038789.1 | 1 | - | Encodes a complete P-type type IV secretion/conjugation system (VirB3–VirB11, TraC, TrbG, TrbM), plasmid replication, stabilization (parA), and multiple toxin–antitoxin systems (fic/doc, VbhA, toxN/AbiQ). |
| NZ_CP071514.1 | 7 | aph(3')-Ia | Aminoglycoside O-phosphotransferase, conferring resistance to aminoglycosides |
| NZ_CP080650.1 | 2 | aph(3')-Ia | Aminoglycoside O-phosphotransferase, conferring resistance to aminoglycosides  It encodes a broad-spectrum class A β-lactamase (TEM-1), which hydrolyzes penicillins and certain cephalosporins, conferring resistance to β-lactam antibiotics. |
| NZ_CP085950.1 | 7 | blaTEM-1 |  |
| NZ_CP119140.1 | 1 | - | - |
| NZ_CP119142.1 | 1 | - | - |
| NZ_CP131721.1 | 2 | blaTEM-1 | Encodes broad-spectrum class A β-lactamase (TEM-1), an enzyme that hydrolyzes penicillins and certain cephalosporins, conferring resistance to β-lactam antibiotics. |
| NZ_JAMRYS010000114.1 | 1 | - | - |
| NZ_JAMRYT010000035.1 | 2 | - | - |
| NZ_JASCYM010000018.1 | 1 | aph(3')-Ia | Encodes aminoglycoside O-phosphotransferase (APH(3')-Ia), an enzyme that inactivates aminoglycoside antibiotics, conferring resistance to antibiotics like gentamicin and kanamycin. |

**Supplementary Dataset 1 Comprehensive virulence gene profiles of 104 *Haemophilus influenzae* isolates.**

This spreadsheet details the presence/absence matrix and sequence identity scores of 162 virulence factors annotated using the Virulence Factor Database (VFDB) across all 104 sequenced isolates in this study.

**Supplementary File Newick Format Core-Genome Phylogenetic Tree**

This file contains the Newick string format of the genome-scale core-genome phylogenetic tree for the 104 *Haemophilus influenzae* isolates.
